# Primary Maxillofacial Large B-Cell Lymphoma in Immunocompetent Patients: Report of 5 Cases

**DOI:** 10.1155/2011/108023

**Published:** 2011-08-24

**Authors:** Ines Velez, Maritzabel Hogge

**Affiliations:** Oral and Maxillofacial Pathology, Nova Southeastern University, 3200 South University, Suite 7393, Fort Lauderdale, FL 33328, USA

## Abstract

Lymphomas of the oral cavity represent 5% of all lymphomas. They usually occur in immunocompromised patients. Lymphoma arising within a single bone, without visceral or lymph node involvement, is known as primary intraosseous lymphoma. It is a rare condition and constitutes 3.1% of malignant bone tumors and 5% of extranodal lymphomas. Primary lymphoma of the jaw is seldom seen and it is often misdiagnosed. Clinically, the manifestations are usually similar to an odontogenic tumor, cyst, or infection. Radiographically it appears as a radiolucent area that may mimic endodontic lesion, periodontal pathology, or odontogenic cyst or tumor. The initial presentation is commonly followed by multiple unnecessary extractions and/or root canal treatments. We present five cases of rare primary lymphoma of the maxillofacial complex, four of them intraosseous.

## 1. Introduction

Lymphomas are malignant neoplasms of lymphoreticular cells. The Revised European-American Lymphoma classification, REAL/WHO system, describes three categories of lymphoid neoplasms based on cell lineage: B-cell malignancy, T-cell/natural killer malignancy, and Hodgkin's lymphoma. Lymphomas and lymphoid leukemias are included in this classification, due to the fact that they are manifestation of the same condition [1].

Non-Hodgkin's lymphomas (NHLs), in contrast to Hodgkin's disease, usually manifest outside of the lymphoid system. Skin, abdomen, lung, central nervous system, and oral cavity are common locations [2]. 

The Waldeyer's ring, located at the entrance of the respiratory and digestive systems, is composed by the palatine tonsils, nasopharyngeal tonsils (adenoid), and lingual tonsils and constitutes the most common place for NHL of the oral cavity. Waldeyer's ring forms part of the mucosa-associated lymphoid tissue (MALT) and presents the first defense line against exogenous aggressors. 

Malignant lymphomas of the oral cavity represent 5% of all lymphomas [3] and are most common among male patients between 50 to 70 years of age [4]. Hematologic malignancies are very often seen in immunocompromised patients [5].

Large B-cell lymphoma (LBCL) is the most common non-Hodgkin's lymphoma. LBCL is a fast growing malignancy that may arise inside or outside of the lymphatic system, usually in the gastrointestinal tract, brain, testis, breast, skin, or thyroid. 

Often the first symptoms of large B-cell lymphoma of the oral cavity are painless swelling of the neck, fever, sweats, and weight loss.

Lymphoma arising central in a single bone, without visceral or lymph node involvement, is known as primary intraosseous lymphoma [4] (PIL). Primary intraosseous lymphoma is a rare condition and constitutes 3.1% of malignant bone tumors [6] and 5% of extranodal lymphomas [7]. The most common PIL is non-Hodgkin's large cell type. 

Primary intraosseous lymphoma of the jaw is seldom seen and it is often misdiagnosed. Clinically, the manifestations are usually similar to an odontogenic tumor, cyst, or infection. Radiographically it appears as a radiolucent area that may mimic endodontic lesion, periodontal pathology, or odontogenic cyst or tumor. This lesion shows destructive but localized behavior. The initial wrong diagnosis is followed by multiple extractions and/or root canal treatments. 

The immunophenotype of diffuse large B-cell lymphoma is variable; therefore it indicates that this comprises a heterogeneous group of tumors. More than 25% of LBCL have a translocation t(14;18), and most of them express bcl-2 with or without a translocation present. Chromosomal rearrangements affecting the bcl-6 gene (regulator of germinal centre formation) at 3q27 are seen in 30% of LBCL extranodal tumors. The t(8;14) also occurs in LBCL as well as in Burkitt's Lymphoma. Mutations and deletion of p53 are common in LBCL. Bcl-6 and Bcl-2 are between the strongest predictors of survival [8]. 

The initial treatment for most patients is known as CHOPR chemotherapy (cyclophosphamide, hydroxydoxorubicin, oncovin, prednisone, and rituxan). Radiotherapy is used in early states for massive tumors. About 45% of patients may be cured using this protocol. 

The International Prognostic Index (IPI) is based on extent of the disease, age, and behavior of the tumor. More than one extranodal site of disease is an indicator of a less favorable prognosis. Most of the patients are in an intermediate category, many of whom will be cured by CHOPR. An extranodal origin seems to be a favorable prognostic factor. Cell variables that may be important in predicting outcome are bcl-2 expression, cell proliferation rate, p53 expression and mutation, and bcl-6 rearrangements [9].

## 2. Report of Five Cases

PIL is an extremely rare condition. All the reported patients were seen in the dental clinics of Nova Southeastern University College of Dental Medicine, between the years 2002 and 2010. NSU Dental clinics receive around 400 patients daily. The Oral and Maxillofacial Surgery Department, Oral and Maxillofacial Radiology Department, and Oral and Maxillofacial Pathology Division, constitute a consultation center for South Florida.

### 2.1. Case 1

A 58-year-old white man was referred to Nova Southeastern University, Oral and Maxillofacial Surgery Clinic for evaluation and treatment of an intraosseous expansive radiolucent lesion of the anterior mandible. Medical history showed sleep apnea and hypertension and the only medication was antihypertensive. No known allergies. 

**Figure 1:**
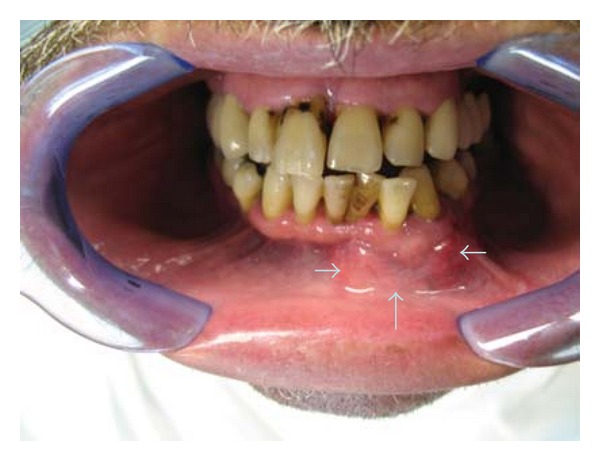
Case 1. Clinical presentation. Swelling of the mandible.

**Figure 2:**
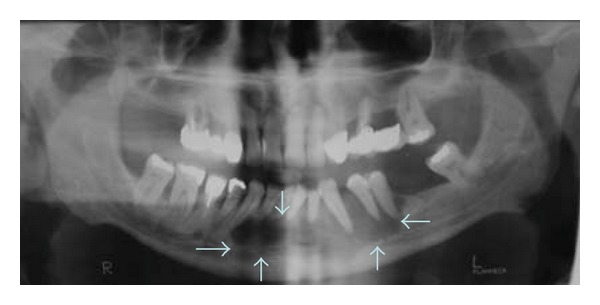
Case 1. Panoramic radiograph showing lytic multilocular lesion: mandible.

Clinically, a painless expansion from buccal to lingual, in the left side of the mandible, covered by normal mucosa and hard upon palpation was noted. The teeth in the area were displaced (Figure 1). Radiographically an osteolytic, well-circumscribed multilocular lesion from the left molar to the right premolar area was seen (Figure 2). Some of the teeth in the area were nonvital, but no root canal treatments were present. The patient did not know the time of evolution. No palpable lymph nodes were found.

With the differential diagnosis of odontogenic keratocystic tumor, ameloblastoma, other odontogenic lesions, and central giant cell tumor, an incisional biopsy was performed under local anesthesia. H&E sections showed a mass composed of uniform, round, hyperchromatic cells with large nuclei and prominent nucleoli. Increased mitotic activity was noted. It was diagnosed as round cell tumor consistent with lymphoma. Flow cytometry showed Kappa monoclonal large B cells, CD3 negative, CD5 negative, CD 10 negative, CD 20 negative, CD 22 positive, CD 23 positive, CD 45 positive, CD 56 negative, Ki67 positive, HLA Dr positive, FMC7 negative, and Bcl-6 and Bcl-2 weakly positive. Final diagnosis was primary intraosseous large B cell lymphoma. The patient is under CHOPR treatment.

### 2.2. Case 2

**Figure 3:**
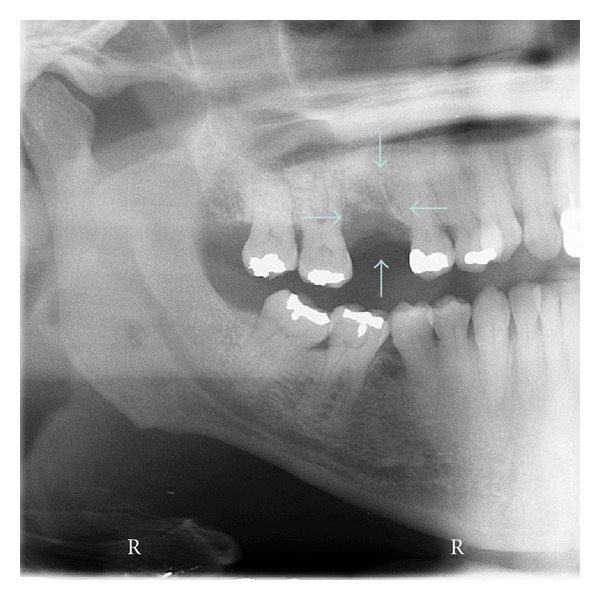
Case 2. Lytic lesion right maxilla.

An African American female, 53 years old, presented to the NSU clinic after a recent extraction of first right maxillary molar. The extraction was performed due to mobility of the tooth. Medical history was no contributory. Expansion of lingual and buccal plates was seen in the area no. 3. No lymphadenopathy was noted. Panoramic radiography showed a lytic lesion in the right maxilla (Figure 3). With the diagnosis of inflammatory lesion of dental origin, an incisional biopsy was performed. Histology and flow cytometry revealed a Large B-cell lymphoma. It was categorized as Primary intraosseous tumor. The patient underwent CHOPR type combination chemotherapy with very good results. Radiotherapy was subsequently performed and she is free of tumor 12 months later. 

### 2.3. Case 3

**Figure 4:**
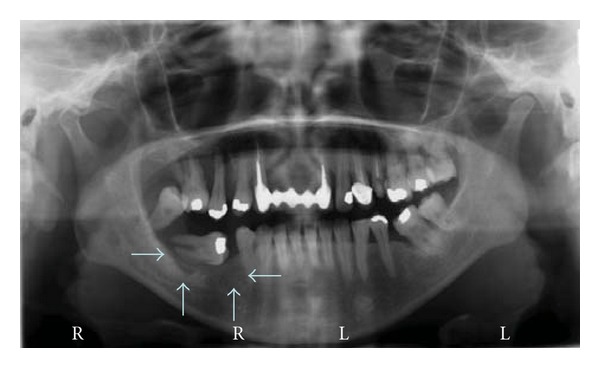
Case 3. Lytic area with displaced floating tooth, right mandible.

A 47-year-old Hispanic female patient, with unremarkable medical history, presented to NSU for diagnosis of a red, 1.5 × 2.0 cms, ulcerated gingival mass in the right mandible; the teeth were freely movable. No other lesions neither palpable lymph nodes were detected clinically. Radiographically: a lytic area with displaced floating tooth around area nos. 30–32 was noted (Figure 4). With the diagnosis of squamous cell carcinoma, an incisional biopsy was done. Erosion of buccal and lingual cortical plates was noted. The initial pathology reported atypical lymphocytic infiltrate and nuclear dust, consistent with lymphoma. Flow cytometry showed monoclonal Kappa B cells, CD5, CD 20, CD22, CD23, CD 45, Ki67, and Bcl-6 and Bcl-2 positive. The final diagnosis was large B-cell Lymphoma. The patient returned to her country of origin.

### 2.4. Case 4

**Figure 5:**
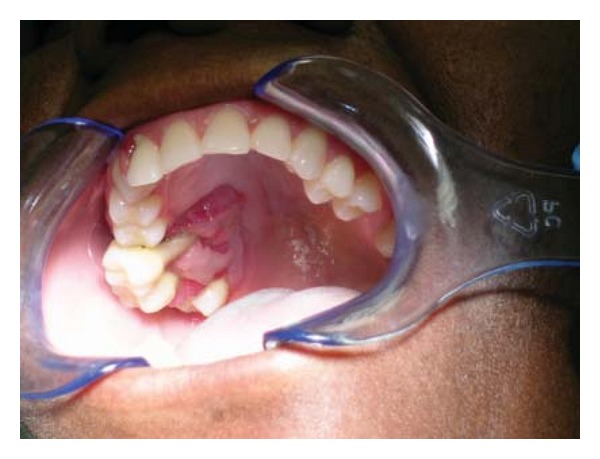
Case 4. Ulcerated, exophytic palatal mass with destruction of the bone and exposed dental roots.

**Figure 6:**
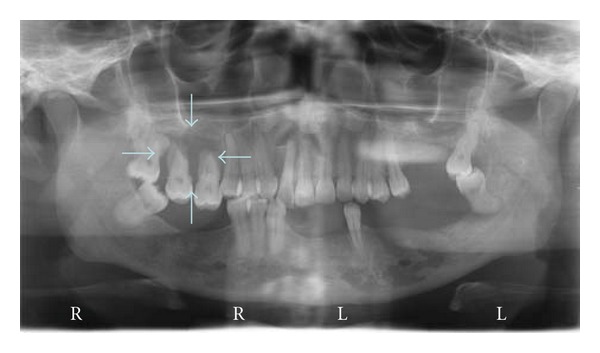
Case 4. Panoramic radiograph showing lytic area with floating teeth. Maxilla.

A 48-year-old African American female, with no contributory medical history, presented to NSU for diagnosis of a red, 6.0 × 3.0 cms, ulcerated palatal mass with destruction of the bone and exposed dental roots, extending to the vestibular area of the right maxilla. The teeth were completely movable. No other lesions and no palpable lymph nodes were present. Radiographically: a radiolucent lesion with floating right maxillary molars was noted (Figures 5 and 6). With the diagnosis of squamous cell carcinoma versus other malignancy, an incisional biopsy was performed. The initial pathology report described atypical round cell infiltrate, consistent with lymphoma. Flow cytometry showed monoclonal Kappa B cells, CD 20, CD 45, Ki67, and Bcl-2 positive. The final diagnosis was large B-cell lymphoma. 

### 2.5. Case 5

**Figure 7:**
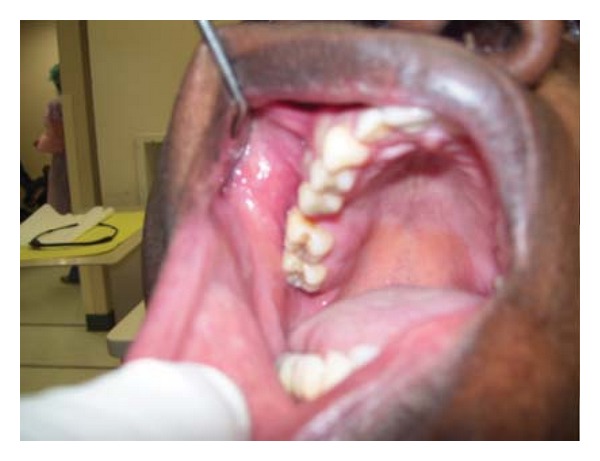
Case 5. Slight swelling of the maxilla.

**Figure 8:**
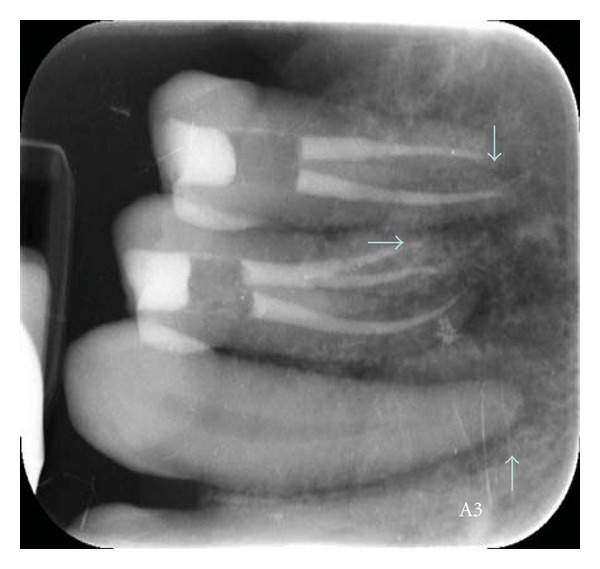
Case 5. Periapical radiograph.

A 48-year-old male “healthy” patient presents to the NSU clinic with slight swelling of the right maxilla (Figure 7). The premolars had root canal treatment and the first molar was extracted previously. The area was tender. No lymphadenopathy was identified. Radiographically; there was a mixed diffuse pattern suggestive of fibro-osseous lesion (Figure 8). After CT Scan and immunohistochemistry, the lesion was diagnosed as intraosseous large B-cell lymphoma. The patient is under CHOPR treatment.

None of the patients had evidence of lymphoma in any other area of the body.

## 3. Discussion

We presented five uncommon cases of large B-cell lymphomas, four of them primary intraosseous, in immunocompetent patients.

Oropharyngeal lymphomas are often complication of HIV-infected persons or immunosuppressed transplant recipients. Hodgkin and non-Hodgkin lymphoma in AIDS patients may appear even under potent antiretroviral therapy (2% of oral neoplasms in AIDS patients) [10]. However, non-immunosuppressed patients of any age can be also affected. Most immunocompetent patients with non-Hodgking lymphoma of the gnathic region are middle aged males. Lymphomas of this area represent 2% of all extranodal lymphomas and can affect both bone and soft oral tissue with the most frequent localization being the Waldeyer's ring. 

Large B-cell lymphoma (LBCL) is the most common type of non-Hogdkin's lymphoma, and it may arise inside or outside the lymphatic system. Often the first symptoms of a large B-cell lymphoma of the oral cavity are a painless swelling, a nonhealing ulcer, fever, sweats, and weight loss. A nonpainful lymph node enlargement and a submucosal lesion in the junction between hard and soft palate are highly suspicious. Often, the oral manifestations are secondary to a more widespread involvement. Primary lymphoma of the oral cavity is not common and primary intraosseous lymphoma in the head area is even rarer and constitutes 3.1% of malignant bone tumors [6]. 

Soft tissue lymphoma of the oral cavity and primary lymphoma of the jaw are often misdiagnosed. Clinically and radiographically, the manifestation is usually similar to squamous cell carcinoma or to an odontogenic tumor, cyst, or infection. 

The initial treatment for most patients is combination of chemotherapeutic drugs. Radiotherapy is used in early states for massive tumors. 

Early diagnosis will improve the prognosis. However, patients older than 60, stages 3 or 4 and several extranodal places of involvement, will have a nonfavorable prognosis. The initial response to treatment is good but this entity shows a prolonged course with remissions and exacerbations. The disease may progress into a leukemia.
